# Adrenal Insufficiency in Young Children: a Mixed Methods Study of Parents’ Experiences

**DOI:** 10.1007/s10897-018-0278-9

**Published:** 2018-07-07

**Authors:** Amy Simpson, Richard Ross, John Porter, Simon Dixon, Martin J. Whitaker, Amy Hunter

**Affiliations:** 1 Genetic Alliance UK, 49-51 East Road, London, N1 6AH UK; 2 Department of Oncology & Metabolism, EU12, The Medical School, University of Sheffield, Sheffield, S10 2JF UK; 3 Diurnal Ltd, Cardiff Medicentre, Heath Park, Cardiff, CF14 4UJ UK; 4 School of Health and Related Research, University of Sheffield, Regent Court, 30 Regent Street, Sheffield, S1 4DA UK

**Keywords:** Adrenal insufficiency, Congenital adrenal hyperplasia, Parent, Psychosocial, Survey, Qualitative, Interviews

## Abstract

Research into adrenal insufficiency (AI) and congenital adrenal hyperplasia (CAH) in children has focused largely on clinical consequences for patients; and until recently, the wider experience of the condition from the perspective of other family members has been neglected. In a mixed methods study, we captured the experiences of parents of young children affected by AI/CAH, including their views on the psychosocial impact of living with and managing the condition. Semi-structured interviews were carried out in the UK and an online survey was developed, translated and disseminated through support groups (UK and the Netherlands) and outpatient endocrinology clinics (Germany). Challenges associated with diagnosis, treatment, support and the future were identified. For UK parents, the diagnosis period was characterised by a lack of awareness amongst healthcare professionals and occurrences of adrenal crisis. Parents reported burden, anxiety and disruption associated with the intensive treatment regimen. Parents adjusted and gained confidence over time yet found delegating responsibility for medication difficult and worried about the future for their child. Access to psychological support and contact with other families was reported as highly beneficial. The findings of the study provide critical context for future studies and for informing how parents and families can be better supported. Prenatal genetic counselling for parents who already have an affected child will include an explanation of recurrence risk but should also focus on providing information and reassurance about diagnostic testing and care for their newborn.

## Background

Adrenal insufficiency (AI) is a rare condition in which the adrenal glands are unable to produce the essential stress hormone cortisol (Charmandari et al. 2014). Before the introduction of cortisol replacement therapy in the 1950s, death in an adrenal crisis and cardiovascular collapse was inevitable. Even with cortisol replacement, patients with AI may undergo adrenal crisis, usually precipitated by infection, and still have twice the mortality of the healthy population (Bancos et al. 2015). AI is classified as primary or secondary; the most common cause for primary AI in children is congenital adrenal hyperplasia (CAH) which results from the inheritance of a mutation in an enzyme that controls production of cortisol. As a result of the low cortisol levels, the pituitary gland overstimulates the adrenal glands causing hyperplasia and oversecretion of androgens, which cause ambiguous genitalia and virilisation in female infants, precocious puberty in both boys and girls and infertility in adults. Patients with primary AI including CAH may also be deficient in aldosterone which is important for controlling salt balance (‘salt-wasting’ CAH is severe and can be fatal; symptoms appear within days or weeks of birth).

A recent review identified that current research in CAH still has a strong emphasis on potential health complications, emergency management and consequences of excess androgen exposure, whilst the management of CAH from the perspective of the family has been neglected (Fleming et al. 2017a).

Previous studies have highlighted a number of challenges associated with living with AI and CAH, particularly in relation to treatment. Although two recent studies focusing on the health-related quality of life and functioning of children with CAH found that they experience few negative side effects (Halper et al. 2017; Sanches et al. 2012), it has previously been argued that health outcomes for adult patients are impaired in part because of the treatment they received in childhood (Arlt et al. 2010; Finkielstain et al. 2012; Hahner et al. 2007; Han et al. 2014; Løvås et al. 2002). Yet, there is a lack of consensus on management approaches (Han et al. 2014), presenting challenges to clinicians (Kim et al. 2012) and patients have reported difficulties such as remembering to take the medication and balancing doses with busy lifestyles (Forss et al. 2012). Patients and families are also responsible for adapting medication to avert adrenal crisis, for example during illness or stress (so-called sick day rules). Whilst treatment for adrenal crisis is highly effective, there is evidence that there is insufficient education about it for patients, and improvements in education could significantly reduce morbidity and mortality (Allolio 2015).

Recent studies have also highlighted inadequacies in information and support provided to parents post-diagnosis (Boyse et al. 2014): in preparing them for managing adrenal crisis (Fleming et al. 2017b); in communicating genetic risk to other family members (Abad et al. 2017); and in learning how to cope, make sense of their new situation and talk to their child (Lundberg et al. 2016). Research studying the parental experience of childhood illness is important as parents often take on the role of mediator and intermediary, particularly between the child patient and the professional (Hummelinck and Pollock 2006); they play a crucial role in how children adjust and manage illness (Aytch et al. 2001); and they underpin the well-being of the family unit (Fisher 2001). In the context of AI/CAH, given the critical nature of the treatment regime, researching the experience of parents is even more fundamental to understanding the full illness experience and identifying how the whole family can be better supported. Existing research points to the importance of information and support particularly in the first 5 years after diagnosis (Fleming et al. 2017b), suggesting that focusing on the experiences of parents of younger children is especially important.

We undertook a mixed methods study focusing on the experiences of parents of young children affected by AI/CAH in the UK, the Netherlands and Germany, including their views on the psychosocial impact of living with and managing the condition. It aimed to (i) capture the experiences of parents through semi-structured qualitative interviews and (ii) further explore the emerging themes and related issues with larger numbers of parents through an online survey. This paper focuses on describing the key challenges experienced by parents and discusses the implications of these findings for healthcare professionals, including genetic counsellors supporting parents after diagnosis and providing prenatal counselling for parents with an affected child.

## Methods

The study used a mixed methods approach in two phases: qualitative semi-structured interviews followed by an online survey designed to capture both quantitative and qualitative data.

### Phase 1: Qualitative Semi-Structured Interviews

Qualitative interviews were conducted with parents in the UK. The interviews were semi-structured, encouraging parents to disclose a full and rich account of their experiences (Smith et al. 2009) at the same time as allowing the researcher to maintain a focus and guide the interview, rather than dictate its direction (Bold 2012). Interview questions were open (‘tell me about…’) and designed to gain a better understanding of parents’ experiences and the psychosocial impact of AI/CAH, from diagnosis to the present. The interview topic guide, informed by findings from the initial literature review, included diagnosis, treatment, impact, information and support and hopes and concerns for the future. A pilot interview was conducted with a parent of a child with CAH—the parent was British but was living outside of the UK at the time. Parents were recruited via the Living with CAH support group in the UK using a purposeful sampling strategy. Purposive sampling is a non-random approach, often used by qualitative researchers to identify and select participants who meet certain criteria. Recruitment material (an advert and information sheet) was disseminated to potential participants via newsletters, members’ news alerts and websites, inviting parents to contact the research team. Parents (both mothers and fathers) of children aged 10 and younger were included in the sample. Those parents whose children were aged over six were asked to focus on their earlier experiences (i.e. their child’s first 6 years of life). Interviews took place between September and December 2014. A small number of the interviews were conducted with both parents present, although typically just one parent chose to take part. The majority of interviews took place within the family’s home and lasted approximately 1 hour.

The interviews were transcribed verbatim by a professional transcribing company who had signed a confidentiality agreement. Transcripts were read and checked for accuracy. The transcripts were then analysed thematically by a member of the research team (AS) with the support of assisted qualitative data analysis software, NVivo 8. A number of strategies can help researchers to achieve analytical depth (Ziebland and McPherson 2006). The coding structure developed over time, as data were coded under both anticipated themes (such as those identified in the literature and those included in the topic guide) and new emergent themes. Following the initial steps in the analysis process, in order to clarify the main topics under each overarching theme, all of the coded data were exported to theme-specific files and a further round of analysis was conducted. A second member of the research team (AH) checked the coding structure and the data coded against it. Research team members AS and AH met regularly throughout the data collection and analysis stages of the study to discuss and clarify emerging themes. Findings were also discussed with a wide range of stakeholders including parents and professionals of different disciplines (via project steering group meetings and conferences), providing the opportunity to consider a variety of insights and interpretations.

### Phase 2: Online Survey

The interview approach allows for participants to talk at length about their journeys and offer a more in-depth account of their experiences, including how their experiences may change or improve over time. Contrastingly, surveys capture a snapshot of views at the time point of completing the survey. We developed an online survey to further explore the anticipated and emerging themes and related issues with a larger number of respondents. This manuscript focuses specifically on items assessing parents’ experiences included in the larger online survey which also examined the impact of CAH/AI on children and use of health services/other types of care.

The survey was disseminated to parents of children with AI (including CAH) under the age of six in the UK, the Netherlands and Germany. The decision to pilot, translate and disseminate the survey in these countries was a pragmatic one based on available resources and existing links with active patient groups and endocrinologists.

The coding framework developed during interview analysis formed the basis of the survey development. Open and closed questions were included to explore a range of key issues including those related to getting a diagnosis, the psychosocial impact of living with the condition, support and awareness, surgery and treating and monitoring the condition. Existing published survey tools were assessed but were found not to meet the aims of the study. Members of the research team (AS, AH, RR, MJW and SD) contributed to the formulation of the research questions. The survey was developed and piloted initially in English with a small number of parents in the UK. Feedback from the parents on the English survey helped to validate content, test parents’ interpretation of questions and to assess the feasibility of completing the survey. The survey was then translated into Dutch and German, checked for language accuracy and piloted by native speakers: a parent in the Netherlands and an endocrinologist in Germany. As such, both also had knowledge of AI and healthcare provision in those countries.

The survey was distributed via patient and parent support groups including the Living with CAH Support Group (UK), the Addison’s Disease Self-Help Group (UK) and the Adrenal NVACP Association (Netherlands). In addition, the survey was advertised via paediatric endocrinologists and their outpatient clinics in Germany. The survey was live for a period of 8 months (July 2015 to February 2016), to allow time for the material to cascade to individuals. Interviewees were not excluded from completing the online survey and due to the anonymity of survey responses it was not possible to determine whether they had. As a result of the recruitment method adopted, it was not possible to calculate a response rate. Due to the sample size, the survey analysis was exploratory and mainly descriptive in nature. Statistical analysis software, SPSS, was used to support the analysis.

## Results

### Phase 1: Interview Findings

In total, 16 interviews took place, with 20 parents (14 mothers and six fathers) of 17 children (nine female and eight male). All interviewees were based in the UK. The majority of parents had children aged 6 or younger at the time of the interview (ages ranged from 3 months to 10 years). Three parents with children over the age of 6 took part and were asked to focus on their experiences during the child’s first 6 years of life.

A number of challenges for parents of children with AI were identified during transcript analysis. These challenges relate to the anticipated themes of the interview topic guide: diagnosis, treating and managing the condition, thinking about the future and practical and emotional support. The themes and subthemes are summarised below and shown with representative quotes in Table 1.

**Table 1:** Interview findings: themes, subthemes and representative quotes

Theme	Subtheme	Quote
Diagnosis	Struggle to convince healthcare professionals	‘I had to really fight for my child for this diagnosis and it’s such an incredibly complex condition’.
	Variation in awareness	‘I think we’ve been very lucky because she was born at an excellent hospital – I know it’s incredibly rare but they might see three cases a year whereas if she was born in a remote hospital they won’t see any cases probably and they won’t know what on earth’s going on’.
	Inadequate communication	‘When the consultant came up, you know, he printed off some sort of page of information off the internet of possible conditions it could be and it was a bit of a scrappy piece of paper with a list of about ten conditions, all of which were awful, and I didn’t want them to be any of those… CAH was on there and it was, sort of, like, well if it’s …not treated it can be fatal, so I remember reading those words… and that wasn’t nice, I would have preferred to have had a nice booklet with a picture of a friendly face on the front’.
	Adrenal crisis before diagnosis	‘…the kind of slap round the face for me and the only thing that upset me about the whole experience was when that doctor said 20 minutes later and he probably would have died… if he’d been in 20 minutes later he might not have made it. And that brought it home’.
Treating and managing the condition	Medicating correctly i. Anxiety	‘I think it’s just practically it’s just the hassle of having to get his medication ready and you can never forget it, you know, you can never forget it. So I think that adds stress. Not buckets of stress but just you’re always thinking right when’s the next time, when’s the next time. And so there’s a, kind of, latent anxiety’.
	Medicating correctly ii. Difficulties preparing doses	‘I think when you’re already a bit traumatised and looking after a new baby, having to chop tablets up into little bits and having to -, we had to dissolve them in a little bit of sterilised water as well when he was tiny and all that sort of faffing around, you could really, really do without. And especially when you’re worrying whether you’ve got the quantities right and you know the tablets you’ve quartered yourself are not accurately quartered and by the time you’ve dissolved it and you’ve left a bit in the pot’.
	Medicating correctly iii. Timing of doses	‘We’re shattered! I am always tired! It’s tiring and [she] I think is tired because even if she goes back to sleep it’s just a disturbed sleep isn’t it and I know it’s not as important for me but I go to bed every night thinking oh God I’ve got to get up at half five and that’s just horrible, because half five…’
	Delegating care	“I mean I’ve got no intention of going back to work because what child minder in their right mind is going to look after him? They’re going to take one look at his sheet and think ‘I don’t think so’.”
	Disruption of family life	‘I think if the hospital visits had been where it was locally that would have made a big difference to me because it was really hard being a new mum, you’ve got a little baby and then you’re having to put them in a car, travel 2 hours to an appointment, have the appointment not knowing what it’s going to be or bring up in there, travel 2 hours back, my little girl had to go into family childcare again so that’s me feeling like I’m abandoning my 4 year old again. And it’s not really what new mums do’.
	A new routine	‘I can’t honestly, don’t even think I’ve ever forgotten to give it to him, you know, we just sort of base it into our routine. I think probably he was so young, it probably helped us and probably helped him, I mean, he’s grown up with the medicine and it’s one of those things where we just sort of got used to it as part of a new routine’.
Thinking about the future	Taking responsibility	‘...but I do have a lot of concern when he’s out of our immediate control when he grows older, you know, we do know stories of students when they go away to university, you know, literally going out getting drunk forgetting all about their medication and dying...’
	Developing normally	‘…you read about all of the horrible things. In my worst case scenario [she’s] going to be... obese, she’s going to be really hairy, she’s going to have psychological issues...’
	Relationships	‘I worry about the future in terms of...She’s never going to be able to have children. She’ll be like –...Never be able to have sex, you know all of like worst things’.
	Life-long medication	‘...when we found out about [his] condition, one of the first thoughts was, you know, he’s not going to be able to do that [travel] so freely, he’s not going to have that freedom because he’s always going to be tied down to getting back somewhere to have his hydrocortisone or his fludrocortisone which needs to be refrigerated’.
	Surgery	‘I worry about the future in terms of...In my worst case scenario...she’s going to have surgery; if she has surgery it will go wrong’.
	Different to peers	‘...she does look different and as she gets older I just think other children and teenagers and everyone worries about their body anyway don’t they and then you’ve got this extra thing to worry about and I think, yeah, it worries me as she gets older, how she is going to deal with it’
	Adult health services	‘I got the impression then that in the UK the care for children with this disease...is excellent, and then they kind of get lost, so once they become adults a lot of people just sort of disappear and presume they just carry on taking the same medication, but they’re not really in touch with the medical professions’.
Practical and emotional support	Sources of support i. Healthcare professionals	‘...you have your trials and tribulations with it, but then you have to take it into the outside world and deal with it on that level and I guess that’s why they use a psychologist because they want to support you in how you cope with surgery, decisions, medicine and all of that’.
	Sources of support ii. Support groups/peer support	‘I went out to meet up with her [mother of an affected boy] and her little boy is six and I have to say that was a godsend doing that, just to see a normal little – I think that might have been a turning point actually from when he became a baby as opposed to just CAH’.
	Gaps in support	‘...it’s the emotional and practical support that you get in that first year that I didn’t get and I managed because he was the third child...and I understood the condition or made myself understand the condition. What really worries me is what happens when there’s a parent who isn’t like that and doesn’t get support, what happens to them?’

#### Diagnosis

The interviews shed light on parents’ experiences prior to and during the diagnosis period. Parents faced challenges associated with clinicians’ low awareness of CAH and some struggled to convince general healthcare professionals (e.g. general practitioners and health visitors) of the significance of their child’s symptoms; they had to ‘fight’ for a diagnosis. There were also reports of differences between professionals and hospitals (particularly in relation to awareness of the condition) and inadequacies in the way that the diagnosis (or potential diagnosis) was given to families. For some parents, their child’s diagnosis was only made as a result of the child going into adrenal crisis.

#### Treating and Managing the Condition

Parents provided a wealth of data about their experience of treating and managing the condition. The following subthemes emerged during analysis.

##### Medicating Correctly

Interviewees argued that much of the burden was associated with having to get the *right dose* of medication to their child, *at the right time*. This was intensified by three factors: the importance of dosing correctly; the practical difficulties in preparing the medications; and the frequency/timing of dosing.

First, parents were aware of the importance of achieving the optimum medication levels to avert both long-term poor health outcomes and the threat of adrenal crisis during illness or stress. Parents not only had to follow routine medication regimes, they were also responsible for recognising when to adapt and adjust the medication during illness or stress (‘sick day’ rules). Second, the way in which the medication (particularly the hydrocortisone) had to be prepared and administered was a contributing factor. Interviewees reported it to be inconvenient and they shared their concerns that their child was not getting the optimum dose of medication each time. Third, the medication was required frequently throughout the day, often starting with an early morning dose.

Interviewees provided detailed accounts of the general disruption and anxiety that they experienced in relation to the medication regime. A lot of the data relate to the constant management required: parents do not get a day off from their caring role. They described ‘another layer’ to parenting and a ‘latent anxiety’.

##### Delegating Care

Interviewees described the concerns and difficulties they faced in delegating treatment responsibility to childcare agencies and schools, including the responsibility to ‘train’ staff and the emotional impact of incidents where medications were not given correctly. Parents reported giving up work in order to delay or manage the transition of care to others, as their child became old enough to join nursery or school.

##### Disruption of Family Life

Frequent medical appointments, often requiring significant travel, are a major source of disruption for families in terms of the time they take up and the impact this can have on siblings and the affected child’s school life.

##### A New Routine

Interviewees provided detailed accounts of their established routines and a narrative of their experiences as they adjusted to their new caring roles. For example, parents talked about the challenges of leaving the supportive environment of the hospital and getting to know and understand their newborn babies. Our findings suggest that skills, knowledge and confidence very much developed over time.

#### Thinking About the Future

Parents reported feeling worried about their child’s future. First, parents had concerns about the possible long-term effects of the condition and medication. They worried about whether their child would grow and develop ‘normally’ and whether they would be able to have children. Second, parents had concerns about how their child would cope with surgery and medication. For example, parents worried about their child being reliant on medication for the rest of their lives and their child taking on responsibility for their own treatment (when they move away from the family home). Parents expressed their concerns about how girls born with ambiguous genitalia would cope psychologically as they get older and as they become more aware of their condition. Third, parents had concerns about the transition from paediatric to adult health services. Finally, parents did not want their child to grow up feeling different from their peers.

#### Practical and Emotional Support

Interviewees talked about various sources of practical and emotional support that they have found helpful, and about times when they recognised their own support needs were not being met. Parents discussed the support in the context of coming to terms with the diagnosis themselves, making complex decisions about surgery and communicating details about the condition to others. For example, parents identified a tension between being completely open and honest about the condition and giving too many details about ambiguous genitalia, surgery and treatment.

Healthcare professionals such as psychologists and specialist nurses are valued for the support they offer beyond the supervising doctors. Being able to make contact with other families, for example through support groups (online forums or organisations that facilitate face to face events), had been a very important—even pivotal—source of support for interviewees. Parents still face unmet needs for practical and emotional support. For example, interviewees cited a lack of access to psychological services and a lack of knowledge of the condition amongst local health teams. Around the time of diagnosis, families can be far removed from networks of family and friends because of the need for care at specialist centres.

### Phase 2: Survey Findings

In total, 57 survey responses were received from parents (the vast majority were mothers (75%, 43/57)) of children aged 6 or below. Similar numbers responded from the UK, the Netherlands and Germany. There were three responses from participants living in other countries: one from the USA, one from New Zealand and one undisclosed. These responses are included in the findings presented below, except where we make comparisons between European countries, where they are excluded. In response to the question ‘what is the cause of your child’s adrenal insufficiency?’, the majority (79%, 45/57) of participants stated CAH; 4% (2/57) reported hypopituitarism; 2% (1/57) reported autoimmune adrenalitis; 2% (1/57) did not know the cause of their child’s AI; and 14% (8/57) selected ‘other’. The majority of those with CAH (91%, 41/45) reported that their child had been diagnosed with classic salt-wasting CAH. Of the respondents’ affected children 31/57 (54%) were male, 25/57 (44%) were female and one child was registered as intersex/indeterminate gender on their birth certificate. The survey results reported here relate to those themes and subthemes identified in the interview phase and outlined above. Because of the small numbers of respondents, analysis is restricted to descriptive statistics. However, where the data suggest an interesting contrast (for example between countries) then this is discussed.

#### Diagnosis

Data shows that diagnosis was made quickly in the majority of cases (see Table 2), with 79% (45/57) reporting that their child was diagnosed before they were 2 weeks old. Yet differences across the three countries can be observed in relation to the diagnosis experience.

**Table 2:** Survey responses: views in relation to diagnosis, medication and support

To what extent do you agree with the following statements?	Strongly agree	Agree	Neither agree nor disagree	Disagree	Strongly disagree	I don’t know/not applicable
Views in relation to the diagnosis period (*n* = 54)						
My child’s diagnosis was made quickly, %	48	28	7	6	11	0
We struggled to get access to specialists with the appropriate knowledge and expertise, %	19	20	9	15	35	2
I left the hospital/appointment feeling well informed about the condition, %	33	43	9	11	4	0
Views in relation to medication (*n* = 44)						
I have been equipped with the appropriate skills and knowledge to manage my child’s condition, %	36	50	7	5	2	0
I do not understand how the medication works, %	4	0	7	48	39	2
I know how to adapt my child’s medication in times of illness, %	57	41	2	0	0	0
I feel confident that I could respond appropriately in an emergency situation (e.g. an adrenal crisis), %	32	43	14	4	0	7
I am happy for my child to be cared for by others, %	23	23	13	8	25	8
I do not trust others to take responsibility for my child’s medication, %	16	11	25	37	9	2
Views in relation to practical and emotional support (*n* = 47)						
I receive consistent information and advice from health professionals, %	15	30	21	19	6	9
Medical professionals have a good awareness of the condition, %	25	32	19	9	13	2
I have been able to access all of the support and information I need, %	30	36	19	9	4	2

None of the children of the respondents living in the UK were diagnosed by newborn screening, whereas 70% (14/20) of those living in the Netherlands and 50% (9/18) of those living in Germany had received the diagnosis this way. In addition, the most common initial symptoms or complications experienced by the children prior to diagnosis were ambiguous genitalia (28%, 16/57) and dehydration/salt loss (25%, 14/57), and a quarter of respondents (25%, 14/57) reported no symptoms or complications prior to diagnosis. Those who reported no symptoms or complications were exclusively from non-UK countries. In contrast, 38% (6/16) of parents living in the UK reported that adrenal crisis was their child’s initial symptom or complication, compared to only 10% (2/20) of parents in the Netherlands and no parents in Germany (see Table 3).

**Table 3:** Survey responses: CAH/AI initial symptoms or complications, by country

Initial symptoms or complications (frequency)	UK	Netherlands	Germany	Other	Total^a^
Ambiguous genitalia	6	5	5	0	16
Dehydration/salt loss	6	3	4	1	14
Not applicable/no symptoms or complications	0	6	7	1	14
Hypoglycaemia	4	2	2	1	9
Adrenal crisis	6	2	0	1	9
Fatigue	4	1	1	1	7
Feeding problems	3	1	0	1	5
Physical development	1	1	1	0	3
Mood/behaviour difficulties	2	0	0	0	2
Total	16	20	18	3	

^a^Respondents were able to tick more than one answer. *N* = 57

A large proportion of survey respondents (76%, 41/54) agreed or strongly agreed that they left the hospital/appointment feeling well informed about the condition. However, 39% (21/54) agreed or strongly agreed that they struggled to get access to specialists with the appropriate knowledge and expertise initially (see Table 2). Respondents living in Germany were much more likely to agree or strongly agree with this statement (64%, 11/17) than respondents in the UK (21%, 3/14) and the Netherlands (26%, 5/19).

#### Treating and Managing the Condition

##### Medicating Correctly

The survey identified that the most common form of treatment was a combination of hydrocortisone and fludrocortisone (78%, 32/41 of survey respondents reported this combination).

A large majority of parents who responded to the survey did feel equipped to manage their child’s condition, they understood how the medication worked and they felt confident to respond appropriately in times of illness and emergency situations (see Table 2). In addition, 75% (33/44) of survey respondents reported that their child’s condition and symptoms were well controlled by their medication regime (scoring 8 or above on a scale of 1 to 10). Yet, almost one third (30%, 13/44) of survey respondents agreed or strongly agreed that the medication had to be given at inconvenient times and a similar number (32%, 14/44) agreed or strongly agreed that their sleeping pattern was affected. The statement ‘I find it distressing giving my child their medication’ elicited a total response of 59% (26/44) disagreeing or strongly disagreeing. However, there appears to be a difference in responses between those living in Germany and those in the UK/Netherlands, with only 19% (3/16) of respondents in Germany disagreeing or strongly disagreeing with the statement.

##### Delegating Care

The survey highlighted a degree of reluctance relating to delegating responsibility for the medication regime to others (see Table 2): 33% (16/48) disagreed or strongly disagreed that they are happy for their child to be cared for by others and 27% (13/48) agreed or strongly agreed that they did *not* trust others to take responsibility for their child’s medication.

##### Disruption to Family or Personal Life

Approximately 33% (16/48) of survey respondents agreed or strongly agreed that their personal time was interrupted as a result of their child’s condition, and 50% (24/48) said they never forget that their child has a chronic illness. Three quarters (75%, 33/44) of respondents disagreed or strongly disagreed with the statement ‘the medication stops us from doing things’. However, the proportion of respondents living in Germany disagreeing or strongly disagreeing with this statement was lower compared to those in the UK and the Netherlands (50%, 8/16). Almost two thirds of mothers (61%, 22/36) reported disruption from work sometimes or often compared to under half of fathers (45%, 5/11). The survey found that patients required care from a variety of healthcare professionals, including hospital doctors, general practitioners and others. In the 57 returned surveys, 23 hospitalisations were reported (with median length of stay for these hospitalisations being 3 days).

#### Thinking About the Future

A large majority (98%, 47/48) of respondents said they sometimes or often worry about their child’s future and almost half (48%, 23/48) sometimes or often worry about their child interacting with others.

#### Practical and Emotional Support

Although survey responses were mixed in relation to consistency of advice received, a small majority of patients agreed or strongly agreed that ‘medical professionals have a good awareness of the condition’ (57%, 27/47) and that ‘I have been able to access all of the support and information I need’ (66%, 31/47) (see Table 2). Fathers were more likely to agree to the latter statement: 82% (9/11) of fathers agreed or strongly agreed compared to 63% (22/35) of mothers. Survey data revealed that generally parents were content with the support they received in relation to their child’s surgery; 75% (12/16) of respondents agreed or strongly agreed with the statement ‘I felt well supported while making decisions about surgery’ and 69% (11/16) agreed or strongly agreed with the statement ‘I have received consistent advice and information about my child’s surgery’. The high number of ‘I don’t know/not applicable’ responses to this question was excluded in the analysis on the assumption that most were ‘not applicable’ because surgery is not required for a significant proportion of patients.

Respondents were asked to choose what types of support they had accessed, beyond the supervising doctors. Only 14% (8/57) reported that they had accessed professional psychological support, and 19% (11/57) had accessed nurse specialists. Larger numbers—but still a minority of respondents—had sought support from patient or parent groups (37%, 21/57) and online networks (39%, 22/57).

## Discussion

This research has demonstrated that parents of young children with AI face particular challenges as the primary caregivers in relation to diagnosis, treatment, support and the future.

### Diagnosis

The data provide insight into the difficulties that families face prior to or during the diagnosis period and differences in experiences across countries were observed. Such differences have not been documented previously, as research focusing on parents’ experiences of managing the childhood condition has tended to recruit participants from a single country (Fleming et al. 2017a). Awareness of specific rare diseases amongst healthcare professionals is inevitably low, but as with many rare diseases, parents’ experiences suggest that there is probably variation across services in terms of awareness of CAH and the ability to make a timely diagnosis.

The absence of a newborn screening test for CAH within the UK’s NHS means that recognition of early clinical symptoms is key. Research has shown CAH is associated with excess mortality due to adrenal crisis (Falhammar et al. 2014; Swerdlow et al. 1998) and therefore obtaining a speedy and accurate diagnosis is crucial. Many parents felt they had to fight to get the attention of doctors and there was a lack of knowledge about the condition amongst professionals, but this seemed to have more impact in Germany. It is unclear why this should be the case in Germany particularly, but it is of note that a survey of the status of the paediatric endocrinology subspecialty undertaken by the European Society of Paediatric Endocrinology in 2017 showed that both UK and Netherlands had well-established subspecialty training and recognition, whereas Germany had no approved subspecialty (Lebl et al. 2018).

### Treating and Managing the Condition

The interview findings from the UK described at length parents’ experiences in relation to the burden, disruption and latent anxiety associated with the importance of dosing correctly. Parents experienced a conflict between the pressure to medicate at the right dose, and the requirement for them to (for example) crush tablets of a larger dose, which is the general practice in the UK. In the Netherlands and Germany, common practice is for pharmacists to prepare the medicine. Such differences in practice may in part explain the variation of responses by country which are suggested by the data. At the time of data collection, and until very recently, there was no licenced treatment for young patients hence the requirement to adapt adult preparations. Following a European Commission funded clinical trial, a novel formulation of hydrocortisone has now been developed and approved so that young patients with AI receive child-appropriate doses (http://www.tain-project.org/).

Survey responses arguably presented a more positive parental experience than interviewee accounts in relation to medicating correctly. This could be attributed to the nature of the two approaches to data capture. Interviews captured parents’ journeys over time (i.e. their experiences from the time of their child’s diagnosis to the present day), whereas the survey captured a snapshot of parents’ views at the time of completion of the survey. Almost 90% of survey respondents had children over the age of one, and so their earliest experiences of learning about and adapting to the regime may not be reflected in their responses. The positive experiences reported by parents may also reflect the simple fact that it is possible to manage the condition through medication, unlike many other rare conditions where access to treatments is very limited (Muir 2016; Limb et al. 2010).

The interview and survey data suggest that over time parents develop confidence and knowledge in relation to managing the condition. However, the data also suggest that it is common for parents to face difficulties in delegating care to others (such as staff in childcare settings or schools), further adding to the burden they experience. These findings are also consistent with previous research focusing on parents’ management of adrenal crisis carried out in the USA by Fleming et al. (2017b). The findings have highlighted the importance of supporting parents to better manage the delegation and transition of care to these individuals.

The data demonstrate that parenthood is impacted to a varying extent by the medical needs of affected children, and this can be manifested in disruption to sleep, to daily routines, to personal life and employment. Such disruption is not surprising given the nature of the care giving role—taking responsibility for an intensive medication regime and attending regular medical appointments. Due to the rarity of the condition, families are likely to be required to travel some distance to access specialist care and treatment. Again, the survey responses were more positive in this respect than the detail that emerged during interviews might have predicted. The survey findings are consistent with the results of a questionnaire study on the functioning of children with CAH and their parents in the Netherlands which found that experienced burden of the condition is low and as children grow older, parents become less afraid of adrenal crisis (Sanches et al. 2012). Again, this might be attributable to the adjustment and coping mechanisms that parents adopt over time whereas the disruption is likely to be more strongly felt in the immediate period post-diagnosis which is a period represented in the interview data but less so in the survey data. In addition, appointments with specialists may be more frequent during this time in order to establish appropriate care plans, and parents may be more likely to seek advice from professionals about their child’s symptoms and the appropriate course of treatment during times of stress and illness.

### Thinking About the Future

Parents reported concerns they have for their child in the future and this finding was consistent across the interview and survey data. As with other rare diseases, parents worry about their children facing health challenges as they grow up, feeling different to their peers (especially at school), and about whether the transition to care provided by adult health services will be effective (Muir 2016; Limb et al. 2010). This latter concern is borne out by an assessment of a UK endocrine centre which found that half of all young people with CAH referred to the specialist adult service between 3 and 10 years earlier were no longer attending (Gleeson et al. 2013).

### Practical and Emotional Support

Interview and survey data around the support received from ‘medical professionals’ (as a general description) were consistent in that both gave a mixed review about of the levels of knowledge about the condition. It is an unavoidable feature of rare diseases that specialists are scarce, and the lack of knowledge amongst professionals providing health services locally is a common challenge for rare disease patients (Limb et al. 2010).

Interviewees reported positive experiences with psychology support services, but the survey data show that very few families had access to this kind of support; parents’ concerns revealed in the interviews are wide ranging and a source of anxiety and demonstrate the need for parents (not only patients) to be better supported psychologically to come to terms with the diagnosis, make difficult decisions about surgery and to communicate with others about the condition. This finding is consistent with other research with parents in the UK and Sweden which concludes that parents face diverse challenges, requiring support which addresses other skills beyond managing the condition (Lundberg et al. 2016).

Interviewees reported the value of connecting with other families to share experiences and practical advice. Perhaps surprisingly, the survey data indicate that a minority of participants in this study access this kind of contact and support despite most participants being recruited via patient organisations. As such, a case could be made for increasing the reach of such peer support activities. In part, this finding may also reflect how parents’ confidence and ability to manage their child’s condition develops over time.

In light of these gaps in support provision, there is some reassurance in the finding that two thirds of survey respondents felt that they had been able to access all the support and information they need. Interestingly, a higher proportion of fathers compared to mothers felt they had been able to access all of the support and information they required. Mothers are more likely to be at home with their child during maternity leave and as such are more often the primary caregivers responsible for daily monitoring and treatment of the condition during the early months of their child’s life. In addition, higher proportions of mothers reported disruption to their work. Tailored support should be provided for new mothers in their caring role and in helping them transition back to work when they are ready.

### Implications for Healthcare Providers

A number of the findings have implications for healthcare professionals. In the UK, the lack of a screening programme meant 38% of newborns presented with an adrenal crisis, which was distressing for the parents and is likely to have had a long-term impact on their view of the condition. Ironically though, this may have helped them to access specialists sooner than parents in Germany. It is important in healthcare systems that awareness around adrenal crises is improved and that there is a pathway for rapid referral to clinicians with experience in treating adrenal failure. Genetic counsellors have a role in talking with families about the recurrence risk for future children and previous research has also highlighted the importance of their role in supporting parents to communicate with other family members following diagnosis (Abad et al. 2017). Such support may be crucial in identifying others at risk and ensuring early diagnosis.

The study identified that although parents gain confidence and adjust over time, they face particular challenges following a diagnosis. Good support and communication with parents during the diagnosis period is important to start to equip them with the confidence, knowledge and skills they will need as primary carers of a child with AI. For parents of a child with AI who are expecting or planning another baby, genetic counsellors have an opportunity to ameliorate concerns the parents may have around management of a newborn. Sensitive handling of these discussions will need to take into account the early experiences of the parents with their existing affected child, which may have been traumatic. They can work with parents to ensure they understand the timing and nature of the diagnostic tests that will be carried out with their newborn, as well as addressing questions about ongoing care.

Parents were concerned with the challenge of delegating care and understanding what the future held for their child. These concerns need to be addressed by all healthcare professionals but especially through informed psychological support which should be provided to both child and parents throughout childhood, particularly in the period post-diagnosis. More specifically, psychosocial support is required for those families whose child is born with ambiguous genitalia and those faced with complex decisions about surgery. A multidisciplinary, holistic approach, is required to meet the needs of these families.

A challenge for any congenital chronic disease is the impact on the family of frequent hospital visits as well as the requirement to take complex medication and this was reported in this study. Healthcare professionals need to optimise their service to take into account the needs of the family, and treatment regimens need to be as simple as possible and supported by training to reduce parental anxiety around preparation and dosing.

### Study Limitations and Strengths

Survey response numbers are small so the quantitative analysis was restricted to descriptive statistics. However, together the two approaches triangulate the data and provide a more detailed insight into experiences than studies which employ a single method. The mixed methods approach also offers a better understanding of how parents’ experiences might change over time. The survey is a new instrument, based on our interview findings. As such, statistical comparisons cannot be made with research with other populations. The majority of participants were recruited via support organisations which may be considered to bias the findings. Further research is required to reach a wider range of families in order to guide development of resources and support for all families.

The key value of our study is that it provides an insight into the wider impact of the condition, beyond a clinical and biomedical focus, in three countries using a mixed methods approach. The content and focus of the survey were guided by the themes that emerged from the voice of interviewees in phase 1. Researchers have claimed that the views of those affected by rare conditions have been neglected within research (Huyard 2009) and little is known about the wider impact on families of children with rare diseases (Dellve et al. 2006). Such an approach can provide insights into the social context in which participants live (Shah and Priestley 2011) and can identify unmet needs and issues within health services and ultimately lead to improvements (Thorne et al. 2002; Ziebland et al. 2013).

The study complements and builds on other research with parents of children with AI/CAH. Given that previous work has identified that parents of children under five feel less able to manage the condition than those with older children (Fleming et al. 2017b), the study offers a valuable perspective by focusing on the experience within the first 6 years of parenthood and provides key insights across four key themes: diagnosis, treatment, support and the future. Previous studies have also been limited in their methods—this mixed methods study recruited parents from both support groups and endocrinologist clinics across three different countries offering a broader viewpoint.

### Future Research

Using the survey tool developed in this study, future research should focus on capturing the experiences of patients and families using the health systems of other countries, and with those who may be less engaged with support organisations, to guide the development of better support for families. Given the importance of good daily management of AI/CAH throughout life, further research is needed around the experience of patients and families during transition of care from parents to other adults (such as in childcare settings); from parents to self-management by the maturing patient; and from child to adult health services. Such research could have an important impact on improving long-term health outcomes.

## Conclusions

This study provides a unique and detailed insight into the impact of living with and managing AI, from the perspective of parents of affected young children in three European countries. It shows that although parents do adjust and gain confidence as caregivers over time, they face a particular burden which is associated with challenges during the diagnosis period; managing and treating the condition; thinking about their child’s future; and access to practical and emotional support. The burden on parents could be reduced, and potentially the health outcomes for their children improved, by raising awareness of the condition amongst healthcare professionals; developing treatment regimens which are easier and less disruptive to administer; and providing better practical and emotional support, including peer-to-peer and professional psychological support. The findings of the study reinforce the need for genetic counselling to address the issue of diagnosis sensitively when parents are planning or expecting further children.

## Abbreviations

*AI*: Adrenal insufficiency
*CAH*: Congenital adrenal hyperplasia
*NHS*: National Health Service
